# *spa* Types and Staphylococcal Enterotoxin Production of *Staphylococcus aureus* Isolated from Wild Boar

**DOI:** 10.1007/s00248-023-02236-4

**Published:** 2023-05-09

**Authors:** Sylwia Banaszkiewicz, Aleksandra Tabiś, Bartosz Wałecki, Karolina Łyżwińska, Jarosław Bystroń, Jacek Bania

**Affiliations:** Department of Food Hygiene and Consumer Health Protection, Wrocław University of Environmental and Life Sciences, Wrocław, Poland

**Keywords:** *Staphylococcus aureus*, Wild boar, Staphylococcal enterotoxins, *Spa* typing

## Abstract

Little is known about the structure of *S. aureus* population and the enterotoxin gene content in wild boar. In 1025 nasal swabs from wild boars, 121 *S. aureus* isolates were identified. Staphylococcal enterotoxin (SE) genes were identified in 18 isolates (14.9%). The *seb* gene was found in 2 *S. aureus* isolates, *sec* in 2 isolates, the *see* and *seh* genes were found in 4 and 11 isolates, respectively. The production of SEs was evaluated in bacteria grown in microbial broth. Concentration of SEB reached 2.70 µg/ml after 24 h and 4.46 µg/ml at 48 h. SEC was produced at 952.6 ng/ml after 24 h and 7.2 µg/ml at 48 h. SEE reached 124.1 ng/ml after 24 h and 191.6 ng/ml at 48 h of culture. SEH production reached 4.36 µg/ml at 24 h and 5.42 µg/ml at 48 h of culture. Thirty-nine *spa* types were identified among *S. aureus* isolates. The most prevalent *spa* types were t091 and t1181, followed by t4735 and t742, t3380 and t127. Twelve new *spa* types, i.e., t20572‒t20583 were identified. The wild boar *S. aureus* population was shown to contain previously identified animal/human-associated *spa* types and *spa* types not identified in humans or animals. We also indicate that wildlife animals can be a significant reservoir of *see*-positive *S. aureus*.

**Supplementary Information:**

The online version contains supplementary material available at 10.1007/s00248-023-02236-4.

## Introduction

*Staphylococcus aureus* is considered one of the most common opportunistic pathogens of humans and animals. This bacterial species is responsible for a wide range of infections, from superficial skin and tissue infections to serious, life-threatening diseases such as pneumonia and sepsis [1]. Furthermore, staphylococcal enterotoxins (SEs) produced by many staphylococcal strains are a cause of staphylococcal food poisoning (SFP) which makes *S. aureus* an important foodborne pathogen [2].

*S. aureus* can colonise both animals and humans. Due to changes in the natural environment, previously isolated ecological niches are now frequently overlapping [3]. It was shown that specific staphylococcal lineages have passed from one host to another and that humans can act as a source of pathogenic strains of *S. aureus* that affect animals. *S. aureus* belonging to clonal complex 5 (CC5) was shown to pass from human to poultry [4] and CC398 from human to pig [5] as well as other livestock and companion animals [6, 7]. The adaptation of *S. aureus* to a new niche changes many of its properties, including those involved in pathogenesis. For a number of clonal lineages host specificity is less pronounced and therefore adaptation to new hosts is more frequent (e.g. ST130, CC398) [8]. Therefore, animals can serve as a source of new altered pathogens for humans [9].

Very little is known about the occurrence and genetic diversity of *S. aureus* in the wild boar population. Meemken et al. [10] concluded that *S. aureus* is a rare coloniser of wild boars, as this pathogen was isolated from 6.8% of the investigated animals. Among these isolates, *spa* types t1181, t6782, and t6384‒t6386 were identified. According to Porrero et al. [11] 17.6% of the wild boar population carry *S. aureus*. The isolates belonged to 36 *spa* types, the most common of which was t3750. In these studies, no CC398 *S. aureus* isolates were found, suggesting a low frequency of this clonal complex in wild boars. However, in 2017 the first isolation of CC398 from a wild boar was reported [12].

Ramos et al. [13] obtained 57 isolates of *S. aureus* from 177 nasal swabs from wild boars from Portugal. The most prevalent *spa* type was t11502 with 37 isolates, and the presence of t011 and t034 isolates that are typically associated with livestock was found in 9 and 8 isolates, respectively.

Even less is known about the enterotoxigenic potential of wild animal–derived *S. aureus*. In a study by Seinige et al. [14], 36.9% *S. aureus* cultures were found in 111 wild boar nasal samples. The researchers screened the isolates for the presence of staphylococcal enterotoxin genes (*sea‒see* and *seh*). The only enterotoxin gene found was *seh*. It was present in 11 isolates, 5 of which belonged to the t127 *spa* type.

In this study, *spa* typing was used to investigate the genetic diversity of *S. aureus* isolates obtained from wild boars in Poland. The enterotoxigenic potential of wild boar *S. aureus* isolates was investigated. *S. aureus* was tested for the *sea*‒*see* and *seh* genes encoding SEs, which are considered the most important food safety hazards.

## Methods

### Isolation and Identification of *S. aureus*

One thousand twenty-five nasal swabs were taken from wild boars (*Sus scrofa*) at a game collection point in north-west Poland during the years 2014‒2017. The swabs were taken by placing a sterile cotton swab approximately 10 cm into the nares. Individual swabs were placed in 20 ml of Giolitti-Cantoni broth (Merck, Germany) and cultured statically for 24‒48 h at 37 °C. Subsequently, cultures were streaked on Baird-Parker agar (Merck, Germany) for further selection of staphylococcal isolates and incubated for 24‒48 h at 37 °C. Single colonies displaying the characteristic morphology of *S. aureus* were transferred to 5 ml of BHI broth (BTL, Poland) and cultured at 37 °C with agitation for 18‒24 h. Species identification was confirmed by PCR, using primers for the *nuc* and *clf* genes, encoding thermonuclease and clumping factor, respectively, as previously described [15]. One *S. aureus* isolate per sample was taken for further analysis. The isolates were stored in BHI medium with 15% glycerol.

### Genomic DNA Extraction

Two-millilitre aliquots of an overnight bacterial culture in BHI broth (BTL, Poland) were centrifuged at 12,000 × *g* for 5 min. The bacterial pellet was resuspended in 150 μl 0.1 M Tris–HCl buffer, pH 7.4, containing 2 units of lysostaphin (A&A Biotechnology, Poland), and incubated at 37 °C for 30 min. Then 15 µl of 10% SDS was added and incubated at 37 °C for 10 min, followed by the addition of 200 µl of 5 M guanidine hydrochloride solution and incubation for 10 min at room temperature. DNA was extracted by phenol and chloroform, precipitated by ethanol, dissolved in 50 μl of UltraPure™ Distilled Water (Thermo Fischer Scientific Inc., USA) and stored at − 20 °C.

### Detection of Staphylococcal Enterotoxin Genes

*S. aureus* genomic DNA was screened for the *sea*–*see* and *seh* genes encoding the SEs being the most important food safety hazards. The detection of staphylococcal enterotoxin genes *sea*–*see* was performed according to Sharma et al. [16]. Detection of the *seh* gene was performed as described previously [15].

### *S. aureusspa* Typing

For all *S. aureus* isolates, *spa* types were determined using the method described by Harmsen et al. [17]. PCR amplicons were sequenced (Genomed, Poland). For *spa* typing and cluster analysis of *spa* types with the minimum spanning tree algorithm, Ridom SeqSphere + software was used [18]. *spa* types shorter than 5 repeats were excluded from the analysis. Where possible, isolates were assigned to MLST clonal complexes (CCs), based on information available in the PubMLST database (https://pubmlst.org/) and literature resources.

### Bacterial Growth Conditions and Sandwich ELISA

For selected enterotoxigenic isolates representing different *spa* types, a growth curve and enterotoxins production using ELISA were determined. Isolates in which enterotoxin genes were identified were subtyped using RAPD-PCR according to Louws et al. [19], then within enterotoxigenic isolates representing a given *spa* type, all isolates with different RAPD profiles were selected for ELISA. A single colony was inoculated in 5 ml of BHI and incubated overnight at 37 °C. Subsequently, the cultures were diluted to OD_600_ = 0.03 in 5 ml of BHI broth and incubated for 48 h with cell count and ELISA assay after 24 and 48 h. Bacterial cells were quantified by plating serial dilutions of the culture on BHI agar. Sandwich ELISA for SEC and SEH was performed as previously described [15], and recombinant SEC and SEH produced as previously described [15] served as controls. The antiserum for the detection of SEB was purchased from Acris (Herford, Germany), and the antiserum for SEE was purchased from Abcam (Cambridge, UK). Sandwich ELISA for SEB and SEE was performed as previously described [15]. Purified SEB (Sigma-Aldrich, USA) and recombinant SEE (Abcam, UK) served as controls. The concentration of enterotoxins in samples was determined using a four-parameter logistic curve. Data analysis was carried out using GraphPad Prism software (GraphPad Software Inc., USA).

## Results

### Identification of *S. aureus* in Wild Boars and Staphylococcal Enterotoxin Genes Content

In 1025 nasal swabs from wild boars, 121 *S. aureus* isolates were identified. Genes of at least one SE were found in 16 isolates (13.2%). The *seb* gene was found in 2 *S. aureus* isolates (1.7%), *sec* in 2 isolates (1.7%), *see* and *seh* gene were found in 4 (3.3%) and 11 (9%) isolates, respectively. One *S. aureus* isolate was found to carry both *see* and *seh* genes (isolate 35WB), and two isolates carried both *sec* and *see* genes (isolates 92WB, and 101WB) (Supplementary Table S1).

### Growth and Production of Staphylococcal Enterotoxins by *S. aureus* Isolates from Wild Boars

In 10 isolates in which SE genes were found by PCR, the bacterial number and staphylococcal enterotoxins production (SEB, SEC, SEE and SEH) was tested using ELISA at 24 and 48 h of culture. The numbers of bacteria ranged from 9.5 ± 0.2 to 9.9 ± 0.2 log CFU/ml after 24 h of culture and from 9.8 ± 0.4 to 10.4 ± 0.3 log CFU/ml after 48 h. Production of SEB was determined in 2 *S. aureus* isolates and accounted for 1.84 µg/ml and 2.70 µg/ml after 24 h and for 2.62 µg/ml and 4.46 µg/ml at 48 h of culture. Production of SEC was determined in one *S. aureus* isolate and accounted for 952.6 ng/ml after 24 h and for 7.22 µg/ml at 48 h of culture. Concentration of SEE was determined in 3 *S. aureus* isolates and ranged from 80.1 to 124.1 ng/ml after 24 h and from 102.4 to 191.6 ng/ml at 48 h of culture. Staphylococcal enterotoxin SEH production was determined in 6 *S. aureus* isolates and ranged from 69.4 ng/ml to 4.36 µg/ml after 24 h and from 188.3 ng/ml to 5.42 µg/ml at 48 h of culture (Table 1).

**Table 1:** Concentration of staphylococcal enterotoxins and bacterial count at 24 and 48 h in BHI cultures of selected *S. aureus* wild-boar isolates

Enterotoxin	Isolate	Concentration [ng/ml] 24 h	Log CFU/ml 24 h	Concentration [ng/ml] 48 h	Log CFU/ml 48 h
SEB	46WB	1840.1 ± 331.3	9.8 ± 0.2	2617.5 ± 418.4	10.4 ± 0.3
	114WB	2697.2 ± 153.1	9.7 ± 0.1	4456.2 ± 919.2	9.9 ± 0.2
SEC	92WB	952.6 ± 155.5	9.8 ± 0.1	7219.0 ± 2,184.7	10.1 ± 0.1
SEE	35WB	116.8 ± 70.9	9.8 ± 0.2	132.5 ± 2.8	10.2 ± 0.3
	92WB	124.1 ± 86.0	9.8 ± 0.1	191.6 ± 28.6	10.1 ± 0.1
	125WB	80.1 ± 4.4	9.7 ± 0.2	102.4 ± 3.2	10.2 ± 0.3
SEH	35WB	448.7 ± 151.3	9.8 ± 0.2	474.0 ± 17.1	10.2 ± 0.3
	41WB	69.4 ± 28.2	9.8 ± 0.2	188.3 ± 7.1	10.4 ± 0.3
	47WB	221.8 ± 99.1	9.5 ± 0.2	498.2 ± 71.1	9.8 ± 0.4
	83WB	526.9 ± 213.1	9.7 ± 0.1	1137.9 ± 106.4	10.0 ± 0.2
	88WB	4364.6 ± 56.9	9.9 ± 0.2	5329.6 ± 565.7	10.2 ± 0.2
	89WB	4239.3 ± 218.5	9.6 ± 0.1	5417.0 ± 379.3	9.9 ± 0.1

### Genotypes of *S. aureus* Isolates from Wild Boars

**Fig. 1:**
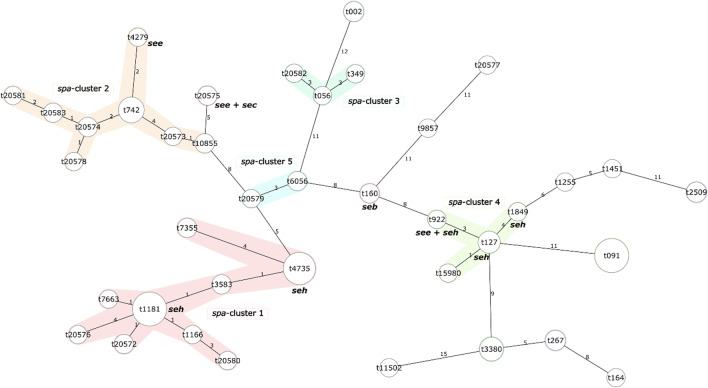
Clustering of *spa* types of wild boar *S. aureus* isolates. Enterotoxigenic isolates were identified using PCR for *sea-see* and *seh* genes. Calculation of similarity between *spa* types and construction of minimum spanning tree was performed using Ridom SeqSphere + . Similarity could not be calculated for *spa* types that contained less than 5 repeats, i.e., t3369, t3424, t3625, t639, and t9909

Thirty-nine unique *spa* types were identified among 121 *S. aureus* isolates (Table 2). The most prevalent *spa* types were t091 and t1181, with 18 isolates each, followed by t4735 with 16 isolates. The* spa* types t742, t3380 and t127 were represented by 9, 7 and 5 isolates, respectively. Sixteen *S. aureus* isolates were assigned to 12 new *spa* types (t20572‒t20583). Based on *spa* type similarity, the isolates were assigned to 5 *spa* clusters (Fig. 1). Similarity could not be calculated for *spa* types that contained less than 5 repeats, i.e., t3369, t3424, t3625, t639, and t9909. Eighty-one out of 121 *S. aureus* isolates were assigned to MLST clonal complexes (CCs) or sequence types (ST) based on literature and PubMLST database. Largest *spa* cluster 1 was formed by 44 *S. aureus* isolates. Thirty-four of them were assigned to clonal complex CC133 (Fig. 1). *spa* cluster 2 was formed by 19 isolates. This cluster contained 9 isolates assigned to CC425 as well as 6 out of 12 new spa types identified in this study. From 3 isolates forming *spa* cluster 3, one was assigned to CC101. Six out of 8 isolates forming *spa* cluster 4 were assigned to CC1. *spa* cluster 5 consisted of 3 isolates, 2 of which were assigned to CC133. All other *S. aureus* were assigned as singletons based on *spa* type similarity. From this, 18 isolates were assigned to CC7, 3 isolates to CC30, 3 isolates to CC97, 3 isolates to CC5, one isolate to CC398 and one isolate was assigned to ST2328 (Fig. 1).

**Table 2:** *spa* types, estimated ST and CC and identified enterotoxin genes in *S. aureus* wild boar isolates.

No.	*spa* type	Number of isolates	Number of isolates harbouring enterotoxin genes				CC/ST
			*seb*	*sec*	*see*	*seh*	
1	t002	3					CC5
2	t056	1					ST101
3	t091	18					CC7
4	t10855	2					
5	t11502	1					
6	t1166	1					
7	t1181	18				3	CC133
8	t1255	1					
9	t127	5				5	CC1
10	t1451	1					CC398
11	t15980	1					
12	t160	2	2				
13	t164	1					
14	t1849	1				1	
15	t20572	2					
16	t20573	1					
17	t20574	2					
18	t20575	2		2	2		
19	t20576	1					
20	t20577	1					
21	t20578	1					
22	t20579	1					
23	t20580	1					
24	t20581	1					
25	t20582	1					
26	t20583	2					
27	t2509	3					CC30
28	t267	3					CC97
29	t3380	7					
30	t349	1					
31	t3583	2					
32	t4279	1			1		
33	t4735	16				1	CC133
34	t6056	2					CC133
35	t7355	2					
36	t742	9					CC425
37	t7663	1					
38	t922	1			1	1	CC1
39	t9857	1					ST2328

## Discussion

*S. aureus* is a ubiquitous coloniser of both humans and animals. Numerous studies have indicated host-specificity of certain clonal lineages of this bacterial species [9]. However, host adaptation of *S. aureus* does not exclude incidental jumps of certain clones to other hosts. It has been shown that livestock-associated *S. aureus* strains may colonise and cause zoonotic infections in humans. In turn, certain human *S. aureus* clones have been shown to pass to animals where they adapt to the new host [8].

To determine the genotype diversity of wild boar *S. aureus* isolates, we applied a widely used molecular typing scheme, i.e., *spa* typing. Analysis of the similarity of *spa* types allowed us to group the isolates into five *spa* clusters. Four of them (clusters 1, 3, 4, and 5) mostly contained already described *spa* types, and in cluster 2, many new *spa* types were included (6 identified in this study out of 9 *spa* types in this cluster). Some of the *spa* types identified could be assigned to known MLST clonal complexes (CCs). The most common CC identified in wild boar *S. aureus* was CC133 (*n* = 36). This clonal complex is considered animal-associated since it was recovered from various animal sources, but not from humans. It has been described as associated with intramammary infections in ruminants, mainly cows [20, 21]. It was also commonly found among *S. aureus* isolates from goats and sheep [22, 23], as well as different species of wild animals, such as Malaysian tapir and birds — e.g., mute swan and Brazilian teal [24]. The second most common *spa* type identified in this study was t091 (*n* = 18), which belongs to CC7. This lineage, mainly associated with humans [25], was already isolated from pigs [26, 27].

Isolates belonging to CC30 and CC398 can colonize multiple hosts. The CC398 clonal complex was indicated to have emerged in humans and spread to livestock [5]. In Europe, *S. aureus* strains from the CC30 and CC398 clonal complexes are prevalent in pigs [28, 29]. CC398 was also found in pigs in Cameroon and South Africa [30]. In the current study, we identified one isolate belonging to CC398 and three belonging to CC30. Sousa et al. [12] reported the first CC398 isolation from a wild boar in 2017.

Nine of the bacterial isolates studied here belonged to *spa* type t742. All of these isolates were included in *spa* cluster 2. The t742 genotype, assigned to CC425 was identified in wild boars [14] and recently in surface waters [31]. Based on the number of hosts in which a given CC was found and the number of isolates identified within the CC, the *S. aureus* clonal complexes were divided into host generalist and host specialist [8]. According to this assumption, CC425 was classified as a host specialist. Human is not a common host for *S. aureus* CC425 [8].

We identified six wild boar *S. aureus* isolates belonging to CC1. The occurrence of this clonal complex in livestock was already observed [26, 32-34], the isolation of these clones has been also reported in humans [25, 35]. Three wild boar *S. aureus* isolates were assigned to CC5. Previous observations by Lowder et al. [4] indicate that most *S. aureus* isolates from diseased and healthy poultry from Belgium, China, Denmark, Japan, the UK, and the USA belonged to the CC5 complex [26, 29, 36]. One wild boar *S. aureus* was assigned to t056 belonging to ST101. This clonal complex is mostly associated with humans [37], but it has also been isolated from cattle [38], non-human primates [37], and rabbits [39]. We identified three wild boar *S. aureus* isolates as members of CC97. This complex is believed to be associated with human and livestock, and *S. aureus* belonging to CC97 was identified in cattle and pigs [40]. The wild boar *S. aureus* population contains previously identified animal/human-associated genotypes, and genotypes not identified in humans or animals.

Most of the enterotoxins identified so far have emetic activity, as demonstrated in experiments conducted in animal models [2]. However, involvement in SFP cases was only demonstrated for a part of known SEs [41]. For this, the screening of wild boar *S. aureus* was focused on SEA-SEE and SEH, enterotoxins whose involvement in SFP was widely confirmed [42, 43]. The *see* gene was identified in an *S. aureus* isolate belonging to *spa* cluster 4 and assigned to CC1, in one isolate belonging to *spa* cluster 2 and in two isolates closely related to this cluster. Two *S. aureus* isolates harbouring the *see* gene were assigned to new *spa* type, i.e., t20575. The *see*-positive *S. aureus* isolate t4279 was assigned to *spa* cluster 2 in which we identified a *spa* type (t742) belonging to CC425, and two isolates of t20575 are closely related to *spa* cluster 2. In turn, one *see*-positive isolate was assigned to t922 *spa* type, which is closely related to t127. t922 was already identified as human, community-associated *S. aureus* [44]. So far, little is known about the occurrence of *see* in *S. aureus*. *S. aureus* carrying this gene was already detected in cows [45], and was listed as the cause of an SFP outbreak in France [46]. Recent research based on whole genome analysis demonstrated that within 883 isolates of *S. aureus*, mainly from human clinical cases and livestock animals, no *see* gene was identified [47]. However, in a recently published whole genome sequence, the *see* gene was identified in *S. aureus* from a wildlife isolate, i.e., European badger (GenBank: CP097571.1). Based on the CP097571.1 genome analysis, we identified the ST of this isolate as closely related to ST425. As mentioned above, this ST belongs to CC425, a host specialist complex that mainly includes animal *S. aureus* isolates. Evidence of an SFP outbreak due to SEE comes from France, 2009 [46]. In this investigation, the total intake of SEE per body was estimated to be 90 ng. Furthermore, the authors quantified SEE in the food sample to be 0.36 to 1.1 ng/g. Our isolates produced SEE at 80‒124 ng/ml at 24 h and 102‒192 ng/ml at 48 h of culture in microbial broth.

Other SEs produced by *S. aureus* from wild boar include SEB, SEC, and SEH. The *seb* gene was identified in two *S. aureus* isolates assigned to the *spa* type t160. This *spa* has already been found in human clinical cases [48], but also in livestock such as chickens and rabbits [49, 50]. The *sec* gene was identified in two *S. aureus* isolates simultaneously carrying the *see* gene. The *seh* gene was found in isolates belonging to CC1 and CC133. Some of the SEs investigated were produced at concentrations exceeding a few micrograms per millilitre of medium. In particular, SEB production reached 2.6 and 4.5 µg/ml at 48 h of culture.

Production of enterotoxins by wild boar *S. aureus* isolates was not assessed previously. We identified wild boar *S. aureus* isolates which carried and expressed the enterotoxin genes *seb*, *sec*, *see*, and *seh.* The *see* gene rarely found in already characterised populations of *S. aureus* was identified in three isolates displaying unrelated *spa* types. Our results suggest that wild boar can be a significant reservoir of *see*-positive *S. aureus*.

## Supplementary Information

Below is the link to the electronic supplementary material.

Supplementary file1 (XLSX 16 KB)
